# A Binder Jet Printed, Stainless Steel Preconcentrator as an In-Line Injector of Volatile Organic Compounds

**DOI:** 10.3390/s19122748

**Published:** 2019-06-19

**Authors:** Xiaolu Huang, Tyler Bauder, Truong Do, Hawke Suen, Connor Boss, Patrick Kwon, Junghoon Yeom

**Affiliations:** 1 Department of Mechanical Engineering, Michigan State University, East Lansing, MI 48824, USA; xlhuang@egr.msu.edu (X.H.); bauderty@msu.edu (T.B.); truongdt.uct@gmail.com (T.D.); hoihohaw@msu.edu (H.S.); pkwon@egr.msu.edu (P.K.); 2 Department of Electrical Engineering, Michigan State University, East Lansing, MI 48824, USA; bossconn@egr.msu.edu

**Keywords:** gas preconcentrators, metal 3D printing, additive manufacturing, stainless steel, thermal desorption

## Abstract

A conventional approach to making miniature or microscale gas chromatography (GC) components relies on silicon as a base material and MEMS fabrication as manufacturing processes. However, these devices often fail in medium-to-high temperature applications due to a lack of robust fluidic interconnects and a high-yield bonding process. This paper explores the feasibility of using metal additive manufacturing (AM), which is also known as metal 3D printing, as an alternative platform to produce small-scale microfluidic devices that can operate at a temperature higher than that which polymers can withstand. Binder jet printing (BJP), one of the metal AM processes, was utilized to make stainless steel (SS) preconcentrators (PCs) with submillimeter internal features. PCs can increase the concentration of gaseous analytes or serve as an inline injector for GC or gas sensor applications. Normally, parts printed by BJP are highly porous and thus often infiltrated with low melting point metal. By adding to SS316 powder sintering additives such as boron nitride (BN), which reduces the liquidus line temperature, we produce near full-density SS PCs at sintering temperatures much lower than the SS melting temperature, and importantly without any measurable shape distortion. Conversely, the SS PC without BN remains porous after the sintering process and unsuitable for fluidic applications. Since the SS parts, unlike Si, are compatible with machining, they can be modified to work with commercial compression fitting. The PC structures as well as the connection with the fitting are leak-free with relatively high operating pressures. A flexible membrane heater along with a resistance-temperature detector is integrated with the SS PCs for thermal desorption. The proof-of-concept experiment demonstrates that the SS PC can preconcentrate and inject 0.6% headspace toluene to enhance the detector’s response.

## 1. Introduction

Monitoring volatile organic compounds (VOCs) plays an important role in many application fields such as environment monitoring, industrial safety, plant health, and human disease diagnosis [1,2,3,4,5,6,7,8]. A state-of-the-art approach to VOC analysis is gas chromatography [9] (GC) coupled with mass spectrometry (MS) [10] or other GC detectors, thanks to their ultrahigh sensitivity, selectivity, and reliability in analyte identification. Solid-phase microextraction (SPME) [11] and adsorbent-based devices [12,13] are used in conjunction with these processes to sample VOCs in the field. Miniaturized versions of GC, which are known as micro-GCs, have received increasing attention over the last two decades [14,15,16,17] due to their increased portability, high response time, and small dead volume. After the first demonstration by Terry et al. [18], numerous efforts have been made to develop the miniaturized GC system using silicon micromachining and microelectromechanical system (MEMS) technologies [18,19]. The key components of a micro-GC system include preconcentrators [20,21,22], separation columns [23,24,25], micropumps [26], microvalves [27,28], and various GC detectors [29,30,31]. Among these components, a miniaturized preconcentrator (denoted as micro-PC) improves the overall detection limit and sensitivity of micro-GC systems with standard sensing technologies by increasing the effective concentration of analytes. Micro-PCs trap target analytes in high surface-area adsorbents during the sampling period, rapidly release the trapped analytes via thermal desorption [32], and then transport them to a testing location. A narrow desorption peak with an increased concentration enhances the separation efficiency of microseparation columns and increases the sensitivity of analysis. Micro-PCs can also function as inline injectors, allowing absorbed analytes to be directly injected into a column or detector without the need for complex plumbing or valves [33].

Numerous developments have been reported on the miniaturization of PCs in micro-GC system. Manginell et al. from Sandia National Laboratories reported on a microfabricated planar PC, incorporating a surfactant templated sol gel adsorbent layer to achieve efficient analyte collection with rapid, efficient thermal desorption [34]. Alfeeli et al. presented a microthermal PC device with 3D structures with a high aspect ratio and coated with a uniform polymer adsorbent film, which has sharp reproducible desorption peaks [12]. Lang et al. developed a micromachined PC consisting of 16 silicon microchannels loaded with Carboxen^®^ 1000 adsorbents to monitor a 100-ppb level of ethylene [35]. Zellers et al. [36,37] developed a sophisticated monolithic and multiple microfabricated PC focuser for the analysis of complex vapor mixtures to improve the thermal desorption performance based on advanced Si-based MEMS technology. Agah et al. reported a new approach of enhancing the adsorption capability of the widely used polymer adsorbent Tenax TA poly(2,6-diphenylene oxide) through its deposition onto a nanostructured template due to the higher surface area of the modified interior surface [38]. Kurabayashi et al. demonstrated a micromachined Si passive vapor PC that was capable of analyzing toluene vapor mixtures at concentrations of ~1 ppm through passive diffusion and featured a thermal desorption efficiency greater than 95% [39]. Finally, Shannon et al. reported a micro-PC with integrated ultrasmall PC volumes and microvalves for fast injection speeds [32].

Most of the fabrication methods for the micro-PCs mentioned above utilized Si-based MEMS technology, in which photolithography and bulk Si etching techniques such as deep reactive ion etching (DRIE) were used to create desired 3D structures. The small-scale device produced by the MEMS technologies has advantages such as low dead volumes and thermal mass, leading to a higher analysis speed and less energy/power consumption. However, they face many of the same challenges associated with other medium to high-temperature gas flow devices produced with MEMS. First, MEMS fabrication typically involves multiple cleanroom-based processes such as photolithography, oxidation, reactive ion etching, bonding, etc., which can be expensive or time-consuming. Furthermore, fluidic interconnections, which are often based on glass capillary tubing, need to be established between micro-PCs and other components when they are integrated in a modular fashion. Typically, these connectors are made of glass capillary tubing, sealed with high-temperature glues. However, this method is susceptible to failure from repeated thermal cycling and lacks a commercial standard. Therefore, developing a simpler and more flexible method of fabricating small-scale PCs with better fluidic interconnection is highly desirable. Here, we propose using metal 3D printing for the fabrication of PCs and demonstrate its applicability.

Numerous microfluidic and microchemical systems have been fabricated using various 3D printing technologies [40,41], but most of them were made of photosensitive polymers, which cannot survive the medium to high temperatures experienced by PCs. While metals or ceramics are more suited for these applications, only a handful of examples have been reported in literature. However, much less work has been reported for microfluidic or microchemical systems made from metal or ceramic 3D printing, which is also known as additive manufacturing (AM). Gupta et al. explored compact titanium alloy chromatography columns with an internal monolithic phase for use with real-time bidirectional temperature modulation capability [42]. Sandron et al. reported coiled planar capillary chromatography columns that were 3D printed in stainless steel or titanium alloy, and showed the potential application of these 3D printed columns in future portable chromatographic devices [43]. To our knowledge, no work has been presented to create 3D printed PCs for GC applications. Moreover, metal devices printed for similar fluidic applications have been fabricated using selective laser sintering (SLS), which requires the removal of any unsintered powders occupying the cavities and channels before use, significantly limiting the size, length, and complexity of any internal geometries.

This report details a novel method of using binder jet printing (BJP), in conjunction with boron nitride sintering additives, to fabricate high-density stainless steel (SS) PCs with sophisticated internal features and robust fluidic interconnectors. The 3D-printed SS PCs, along with the commercial connectors, were tested for gas leakage under high operating pressure. A flexible membrane heater and temperature sensor were integrated with the 3D-printed SS PC for rapid heating with a controlled ramping rate. The PC’s internal cavity was filled with high surface-area adsorbents and tested as an inline injector for trapping a model volatile organic compound by thermally desorbing it at a higher concentration. We also studied the effect of device thermal mass, level of insulation, and heating rate on PC performance. The resulting device can serve as a front-end injector for portable and real-time gas sensing applications.

## 2. Device Fabrication and Experimental Setup

### 2.1. Design of 3D-Printed SS PCs and Fluidic Interconnection

**Figure 1:**
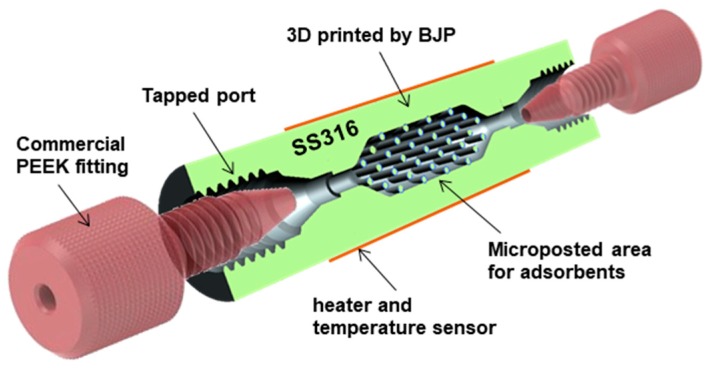
A schematic of the proposed stainless steel (SS) preconcentrator (PC) with commercial PEEK fitting (F-100, IDEX Health; Science Inc.) for fluidic interconnects.

Figure 1 shows the cross-section view of the printed PC with a membrane heater/temperature sensor and commercial fluidic connectors. A 10-32 standard port thread was machined into both ends to accommodate Teflon (1/16” OD), glass capillary tubing (530 μm OD), or PEEK (polyetheretherketone)-based fittings from IDEX Health Science, which provide a gas-tight fluidic connections that can operate under pressure up to 1 MPa and temperature up to 343 °C. The PC has an outer diameter (OD) of 10 mm and length of 38.68 mm. Solid adsorbents (HayeSep D, Sigma Aldrich) were packed into the center of the PC to trap volatile compounds (Toluene). Since the adsorbents are thermally insulating, an array of cylindrical posts (0.75 mm in diameter) were printed to distribute heat more uniformly across the adsorbent bed. The post-gap (separation) is related to the minimum printing resolution as well as the pressure drop and heat conduction.

### 2.2. Fabrication of SS PC via BJP

**Figure 2:**
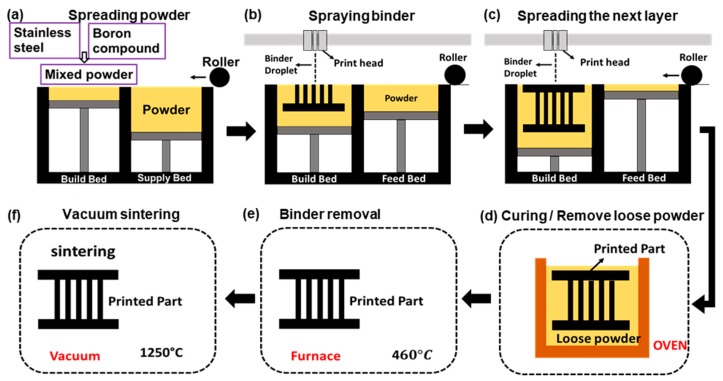
Schematics of the binder jet printing (BJP) process flow: (**a**) delivering and spreading stainless steel (SS) powder from the feed bed to the build bed, (**b**) spraying binder phase in the build bed according to the design, (**c**) delivering and spreading the next layer of SS powder and repeat (**b**) and (**c**) until the part is fully printed, (**d**) curing the part and remove loose powder, (**e**) burn out binder phase in an air furnace, and (**f**) sintering the part in a vacuum furnace.

Samples were fabricated using an ExOne MODEL binder jetting printer (BJP) (X1-Lab, ExOne Inc., Huntington, PA, USA) using spherical stainless steel 316 (SS316) powder (OERLIKON Metco (US) Inc., Troy, MI, USA) and boron nitride (BN) sintering additive (Sigma-Aldrich). Most 3D printers produce 3D geometries by first discretizing them into 2D slices or layers, and then somehow building up each layer atop the previous until the final part is completed. BJP uses an inkjet-like head to deliver a polymeric binder solution to the desired locations of a powder bed. Unlike other printers, BJP operates at room temperature, and does not require a vacuum or shielding gas. The whole process can be broken into several steps, as shown in Figure 2. First, a 3D drawing of the device design (Figure 1) was created using CAD software and imported into the BJP machine. A mixture of SS316 powder (average particle size ~14 μm) and 0.25 wt% BN sintering additive powder (average particle size ~1 μm) was fed to build both the bed and the powder supply bed. A roller uniformly spread the mixed powder from the feed bed to the build bed (Figure 2a). Polymeric binder droplets were deposited onto the designated area of the build bed (Figure 2b). The powder spreading and binder phase printing steps were repeated (Figure 2c) in a layer-by-layer fashion (after each layer, the build bed stage is lowered, while the supply bed stage is raised) until the part was fully printed. Upon the completion of the printing, the build bed along with the printed part was put into an oven to cure the binder phase at 195 °C for 2 h (Figure 2d). After loose powder was removed, the printed part was again put into an oven this time at 460 °C for 2 h to decompose the remaining binder phase (Figure 2e). At this point, the part was fragile, and therefore should be carefully transported to a high-temperature vacuum oven (Model G-3000, Materials Research Furnaces, Allenstown, NH, USA) for sintering at 1250 °C for 6 hours. The vacuum pressure was maintained at 1.33 Pa, better consolidating powder particles and helping to achieve near full-density parts.

### 2.3. Materials Characterization

Scanning electron microscope (SEM) images and electron dispersive x-ray spectroscopy (EDS) for elemental analysis were collected from JEOL 6610LV. X-ray photoelectron spectroscopy (XPS) was also used for additional elemental analysis for light elements and performed in a Perkins Elmer Phi 5600 ESCA system with a magnesium Kα X-ray source at a take-off angle of 45°.

### 2.4. Membrane Heater Fabrication

Instead of using a built-in thermal desorption unit (TDU), which is common in GC, we developed a membrane heater and temperature controller to heat the PCs with reasonable speed and power consumption. A membrane heater was made by sandwiching a serpentine pattern of a thin NiCr wire (40 AWG, Coil Society Online, New York, NY, USA) between two Kapton tapes (50-μm thick, Uline Inc., Milwaukee, WI, USA). The nominal resistance of the membrane heater is around 245.7 Ω. To monitor the temperature, another serpentine pattern of a metal wire with a high temperature resistance of coefficient (TCR) was placed on top of the heater membrane, serving as resistance temperature detectors (RTDs). Nifethal 70 (40 AWG, Coil Society Online, New York, NY, USA) was chosen for this purpose, as its TCR is around 5.25 × 10^−4^/°C, which is high enough to produce sufficient resistance differences upon mild temperature change. The RTD resistances were measured as a function of temperature to verify the TCR values.

### 2.5. Preconcentrator Testing

**Figure 3:**
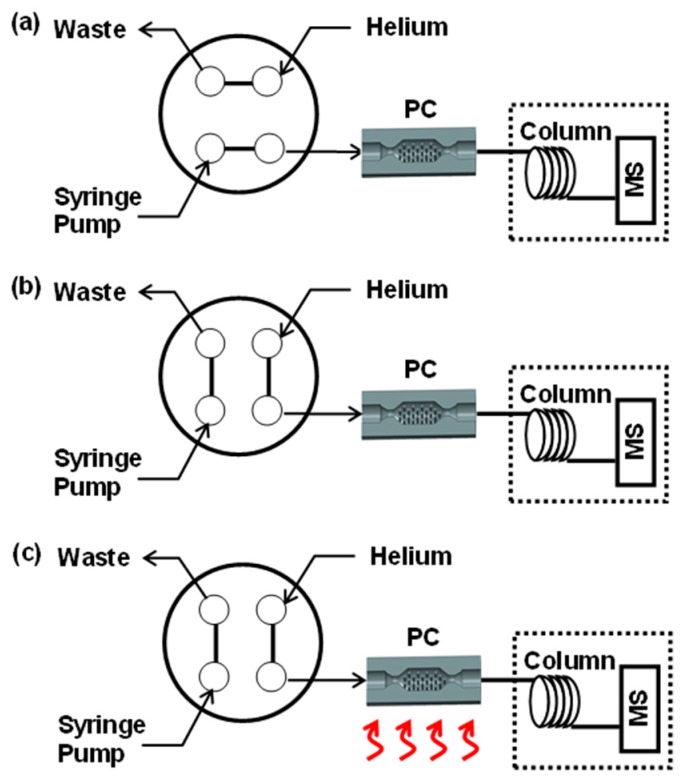
Schematics of sampling and injection steps using a four-port valve: (**a**) in a sampling step, a diluted toluene is trapped in a PC, (**b**) as a valve position is switched, a carrier gas of helium flushes residual toluene in the transfer lines and PC, and (**c**) thermal energy is applied to the adsorbents to release trapped toluene to GC/MS for detection.

The adsorbent material, Hayesep D (100/120 mesh, Supelco, Bellefonte, PA, USA), is a porous polymer with a specific surface area of 795 m^2^/g. First, a small quantity of glass wool was inserted at one end of the device. Adsorbent particles were poured into the PC chamber from the other end with light tapping until it was tightly filled (see Supplementary Figure S1). Glass wool was also inserted at the other end, serving as a placeholder for the adsorbents. Hayesep D is a suitable candidate for trapping volatile organic compounds (VOCs) due to its high surface area. In this study, toluene was selected as a model VOC to test the performance of the PCs. Figure 3 shows the sampling/desorption configuration used for PC testing. In the sampling stage (Figure 3a), a diluted toluene vapor (0.6% of headspace) was charged into a PC using a syringe pump (Harvard Apparatus) through a four-port valve (VICI, Inc., Houston, TX, USA). The outlet of the PC was connected to the mass spectrometer (MS) through an uncoated, passivated column (50-m long) to minimize surface adsorption inside tubing during sample transport. A sampling volume of 10 mL was passed through the PC, so that the adsorbents were charged with toluene. No breakthrough of toluene was observed in the GC-MS instrument (Agilent GC-MS L). The diluted toluene sample was prepared in a Tedlar bag by inserting 6 mL of the headspace of toluene into 10 L of clean, dry nitrogen. In the next step (Figure 3b), the four-port valve switched its position such that a carrier gas of helium (a flow rate of 0.5 mL/min) flushed any residual toluene vapor in the transfer lines and the dead volume of the SS PC, and the baseline signal of the GC-MS instrument (Agilent GC-MS L) was obtained. In the final step (Figure 3c), while the carrier gas was flowing, the SS PC was rapidly heated to the target temperature (180 °C) to thermally desorb the trapped toluene from the adsorbents. These desorbed species were eluted and detected by GC-MS. In order to prevent heated and desorbed toluene from being condensed inside the capillary tubing, the segment of tubing from the SS PC and the GC-MS oven was heated to 110 °C with a custom-made heating jacket. The GC-MS oven was also maintained at 110 °C.

## 3. Results and Discussion

### 3.1. Device Design and Fabrication Results

Several important design and operational considerations for PCs include PC volume (i.e., adsorbent volume and surface area), dead volume, thermal mass, level of thermal insulation, heating rate, etc. First, a PC volume is an enclosed volume occupied by adsorbents that typically possess high surface areas per unit volume. A small PC volume means a low adsorption capacity and is susceptible to analyte breakthrough. However, a large PC volume takes too much time or power to desorb, and can saturate column (or degenerate separation efficiency). Therefore, the PC volume can be tailored to a particular application. Thanks to their small sizes, the microfabricated MEMS PCs have very small PC volumes that are suitable for focusing and injecting into column separators. Conversely, PCs have a relatively large PC volume, because the printing resolution of BJP is limited to a few hundreds of microns; not all that are found in the MEMS PCs can be printed. Our PC chamber volume is around 117.7 mL, and the absorbent mass is 119.9 mg; thus, we consider it useful for an in-line injection application. Secondly, dead volume is another important parameter influencing the preconcentration factor because, if there is any dead volume, the desorbed species become diluted, and PC performance degenerates. In our work, the dead volume is not an issue, as the PC is used as an in-line injector (to be discussed later). Thirdly, the thermal mass and level of thermal insulation affect the heating, cooling, and power consumption rates during operation. Thermal mass should be minimized to increase both heating and cooling rates and decrease power consumption. The wall thickness of the PCs cannot be arbitrarily small, since the BJP parts are known to be porous (even with sintering additives), and a thin wall may result in gas leakage. Therefore, several wall thicknesses were explored to test the effect of thermal mass and wall thickness on the PC’s performance. Finally, the heating rate is an operational parameter that influences the speed of analyte desorption, i.e., peak height and width. In practice, a higher heating rate should provide a higher preconcentration factor for a PC with the same adsorption capacity. SS316 has a higher thermal conductivity (*k* = 13 ~ 17 W/(m·K)) than most polymeric materials (*k* < 0.5 W/(m·K)), and thus spreads the heat from the heating element quicker and more uniformly than the polymeric PCs part. However, the adsorbent materials have low thermal conductivity (in the case of a porous polymer, *k* = 0.01 ~ 0.03 W/(m·K)), so an array of cylindrical posts printed into the PC chamber better distribute the thermal energy.

**Figure 4:**
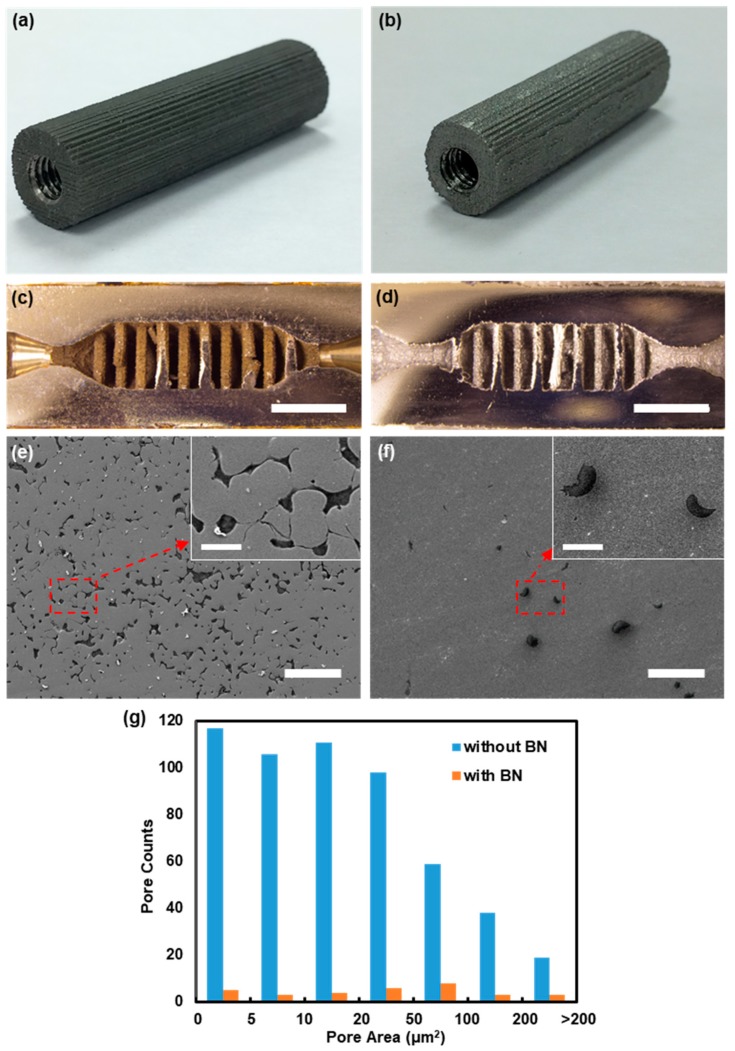
Photographs of the as-sintered preconcentrators (PCs) (10-mm in diameter and 38.68-mm in length) with (**a**) stainless steel (SS) only and (**b**) SS and boron nitride (BN); optical microscope images of the cross-sections of the as-sintered PC with (**c**) SS only and (**d**) SS and BN, scale bar = 5000 μm; SEM images from the cross-sections of the as-sintered PC with (**e**) SS only and (**f**) SS and BN, scale bar = 100 μm, (inset) zoomed-in images of the red-marked area, scale bar = 10 μm; (**g**) histogram of pore areas in the images (**e**) and (**f**).

Figure 4a,b shows the photographs of two printed PCs of the same design: one made of SS316 powder only, and the other made of SS316 powder and BN additives. The PC with SS316 and BN is smaller in size and shinier (more silvery) than one with SS316 only. Prior to sintering, the diameter of the PC was 10.29 mm (note that the designed diameter is 10 mm). After sintering at 1250 °C for 6 h, the resulting diameters were 9.79 mm for the SS316 only and 9.3 mm for the SS316 and BN PCs, respectively. The difference in diameter can be attributed to the sintering additive, BN, which promotes the liquid formation for enhanced sintering. By carefully controlling the amount of BN mixed into SS316, it is possible to control the amount of the liquid phase produced in order to promote sintering at lower temperatures while minimizing the shape distortion. However, reducing the porosity results in more shrinkage. Too much BN can cause an extensive formation of the liquid phase, which can substantially distort the part shape. Similar distortion can occur if the part is sintered at much higher temperatures. In our previous work [44,45], the optimal quantity of sintering additives at various sintering temperatures was determined to maximize the densities of SS420 and SS316 parts without noticeable distortion of the part shape. The degree of reflow and the resulting part distortion were highly sensitive to the sintering temperature. Another reason that the sintering additives can increase the part’s density is a large difference in particle size. The size of BN powder particles is around 1 μm, while that of SS316 powder is around 14 μm. Therefore, when two powder particles are well mixed, the BN powder occupies the interstitial spaces among the SS316 powder, effectively decreasing the porosity and improving the density. The incorporation of BN into SS316 also helps improve the surface smoothness of the fully sintered part [44,45]. The shinier (more silvery) surface of the 3D-printed PC with SS316/BN (see Figure 4b) results from the better surface quality [45].

The densities of the SS316 PC and SS316/BN PC were measured using the Archimedes principle and compared to the bulk SS316 specimen of a known dimension. The specific gravity of the printed part was determined by *W*_s,air_/(*W*_s,air_ – *W*_s,water_), where *W*_s,air_ is the part’s weight measured in air, and *W*_s,water_ is a part weight measured when it is fully immersed in water. Likewise, the specific gravity of the bulk sample was also measured and used to compute the relative density of the 3D printed parts. The relative densities of the SS316-only PC and the SS316/BN PC were measured to be 71.1% and 77.7%, respectively, and both values are significantly lower than what we obtained from our previous works (the cube samples made of SS316 and various amounts of BN had their relative densities ranging between 96–99%) [44,45]. This discrepancy can be attributed to the fine internal features of the PC design that may have trapped air bubbles for the measurement of *W*_s,water_. We repeated the experiment after cutting the PC samples into smaller pieces that no longer have small internal structures, and found that the relative densities were 80.4% and 96.6% for the SS316 piece and SS316/BN piece, respectively.

Meanwhile, the density of the SS PC parts can be indirectly estimated by examining the porosity level from the cross-sectional image analysis. The samples were sectioned and polished using a lapping film with progressively finer silicon carbide grinding papers and polished to 1-µm alumina powder. The optical images of the cross-sectional views of both samples (3D-printed PCs with SS316 only and with SS316/BN) can be found in Figure 4c,d. It is clear from the SEM images in Figure 4e,f that the SS316-only PC is significantly more porous (darker regions) compared to the SS316/BN PC. Four SEM images from various regions of the samples were analyzed using a custom MATLAB script. The script utilizes several built-in functions in the MATLAB Image Processing Toolbox such as adapthresh and bwboundaries, to identify, segment, and quantify the porosity. The code uses adaptive thresholding to identify the dark regions of the image, which are assumed to be pores, binarizes the image, preforms minor binary morphological operations such as closing holes, and calculates the porosity by comparing the number of identified pixels to the overall image size. Then, bwboundaries is used to trace the outlines of identified regions in order to gather information on individual pore size and frequency. Using this method, the porosity was calculated to be approximately 8.05% and 0.92% for the SS-only PC and SS316/BN PC, respectively. If the porosity is simply converted to a relative density, i.e., 91.95% and 99.08% for the SS-only PC and SS316/BN PC, both values are quite larger than the ones obtained from the Archimedes method. The overestimation of the relative density may be related to the limited number of the images and the single cross-sectional plane used in the analysis, failing to capture potential non-homogeneities in the pore distribution of the 3D part.

The porosity level and pore shape are important for microfluidic applications, as it can allow gas leakage through the device walls. While the porosity of 8.05% in a structure may not cause a leakage (in the case of randomly distributed spherical pores), it can be seen in Figure 4e that they are highly elongated and connected. Therefore, one can perceive that there may be a percolation path of gas molecules through the structure even with a relatively low porosity. When BN is incorporated in the SS structure, those elongated pores mostly disappeared (see Figure 4f), and the observed pores were more isolated and rounded, suggesting that a gas leakage is less probable through the structure. Figure 4g presents the pore area distributions of both samples for the same-size imaging area (using Figure 4e,f). It clearly demonstrates that the total number of pores is far fewer for the SS316/BN PC. In addition, there are significantly more small-size pores in the SS316-only PC, but the small-size pores tend to merge favorably when BN is present in the powder mixture.

Finally, elemental analysis was performed in EDS and XPS to reveal the chemical composition of the printed/sintered parts. Table 1 shows the comparison of the chemical composition between bulk SS316 and the printed SS316/0.25%BN part (note that the weight percent data were analyzed from the EDS result, as seen in Supplementary Information Figure S2). The weight percentage of each element measured from the printed part matches well with the bulk counterpart of SS316. Boron was not detected in the printed sample, which is most likely because EDS is not suitable for detecting light elements. Two XPS survey scans were analyzed to reveal the elemental compositions of the SS316/0.25%BN part, as shown in Table 2. One of the scans shows the boron signal and confirms the presence of boron. However, note that the boron concentration is very small, i.e., 0.25%wt, which is on the borderline of the instrument’s resolution (see the raw XPS data in Supplementary Information Figure S3).

### 3.2. Fluidic Interconnect and Leak Test

In order for micro-PCs to function in a chemical analysis system, they need to be connected to other components such as separation columns and sensors. Unless they are monolithically integrated [18], each of these components is modularly integrated and typically connected via polyimide-coated glass capillary tubing [46,47]. The majority of micro-PCs are silicon based, which is brittle and difficult to machine for fluidic connections. Therefore, capillary tubing is attached to the inlets and outlets of silicon-based devices with the help of high-temperature glues or adhesives [36,37]. Such approaches are not sufficiently robust for continuing temperature cyclic operations, and can be susceptible to contamination and large dead volumes. In our case, the printed SS316 PCs are easily machinable; therefore, commercial compression fitting can be used to create leak-tight, robust, and low dead volume connections. It is also important to point out that unlike gluing, compression fittings can withstand much higher pressure operation while the tubing is flexible and easily replaced.

Typically, metal additive manufacturing (AM) techniques including the BJP processing with the addition of sintering additives provide near net shapes of desirable parts, which enables generating threaded holes for compression fittings for the SS PCs. However, the inherent surface roughness of any AM techniques, including BJP, makes a leak-tight seal almost impossible without some amount of post-processing. We have printed PCs out of both SS420 and SS316 powders. However, we found that martensitic SS420 parts are difficult to machine. This was not the case for austenitic SS316 PCs, which remained machinable after going through the same post-printing processes. As a result, all the subsequent PCs were produced with SS316, drilled tapped, and threaded for a 10-32 PEEK fitting. See Supplementary Information Figure S4 for the SS PC connected with the PEEK fitting.

Leak testing was performed on both SS316 PCs, with and without BN sintering additives. With the PEEK fitting and tubing attached, the assembled part was immersed into an isopropyl alcohol (IPA) bath while dry nitrogen was pumped through the assembly, and gas flow would form bubbles in the solution. Figure S5a in the Supplementary Information shows that no bubble was observed (meaning no leakage) when an air pressure up to 500 kPa was applied to the dead end through the SS316/BN PC. Conversely, a significant air leak was observed originating from the walls of the SS316-only PC (see Figure S5b), even when a significantly smaller air pressure of ~64 kPa was applied. This observation is consistent with the porosity levels seen from the SEM images in Figure 4, which could allow the air inside the PC to escape through the walls. The video clips of leakage testing of these two SS PCs are available in the Supplementary Information. It is also important to note that no apparent leak was observed from the PEEK connection area for either SS types. Based on these observations, only the SS316/BN PCs were considered for the rest of the work.

### 3.3. Membrane Heater and RTD Sensor Characterization

**Figure 5:**
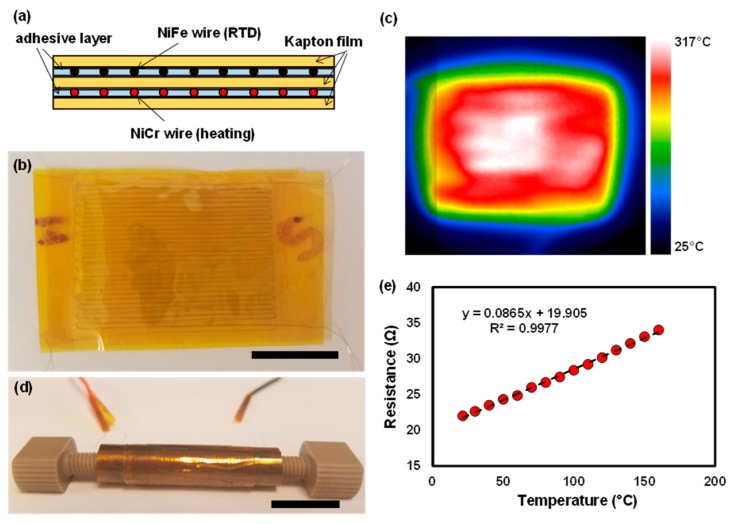
(**a**) A schematic diagram of a flexible membrane heater and resistance temperature detector (RTD), (**b**) a photograph of the fabricated membrane heater and RTD using the Kapton^®^ films, NiCr and NiFe wires; wires were weaved in a serpentine pattern to increase the overall length and resistance, scale bar = 10 mm, (**c**) an infrared (IR) image from a thermal camera (SEEK Thermal Inc.) when 14.6 W of power is applied to the heater, (**d**) a photograph of the SS316/BN PC wrapped with the heater/RTD, scale bar = 15 mm, (**e**) a calibration curve for the RTD sensor.

The release of adsorbed species from the PC is typically accomplished by thermal desorption. In this work, a custom-built heater was fabricated and attached to the PC as a heat source. In order to minimize the form factor (and in turn the associated thermal mass), we have developed a thin, flexible membrane heater that wraps around the SS PC. A heating rate and final PC temperature are important experimental parameters; therefore, a thin, flexible temperature sensor was also developed and integrated to the heater/PC for the feedback control of temperature. Figure 5a shows a schematic of the membrane heater/sensor stacked on top of each other between the thin polyimide membrane (Kapton^®^ tape). The heater layer was made of a serpentine pattern of a thin nickel chrome (NiCr) wire (80 μm in diameter), while the sensor layer was made of the similar serpentine pattern of a thin nickel-iron (NiFe) wire (80 μm in diameter). NiCr has a small temperature coefficient of resistance (TCR, α = 1~15 × 10^−6^ /K), which is suitable for heating application because the resistance of the wire resistor does not vary significantly upon heating. Conversely, NiFe70 has a relatively large TCR value (α = 4000~4500 × 10^−6^ /K), making it feasible to be used as a resistance temperature detector (RTD) because a small change in temperature can cause a measurable response in wire resistance. Figure 5b shows the top–down view of the fabricated heater/sensor stack. The wire was closely weaved in a serpentine pattern with a uniform pitch to increase the total resistance value (resulting in a higher response for RTD) as well as achieve more uniform heating. When 14.6 W of power was applied to the heater, the stack was rapidly heated to 315 °C in 12 s. The uniformity of the temperature distribution across the membrane heater/sensor can be seen in Figure 5c by a thermal camera (SEEK Thermal Inc.). Figure 5d shows the SS PC wrapped with the membrane heater/sensor and assembled with two PEEK fittings. The thermal conductivity of PEEK is much lower than SS, so during the heating period, we assume that the conduction loss to the fittings is not as significant as the convective loss through the outer heater surface. Finally, the resistance (*R*) of the RTD sensor was measured as a function of temperature (*T*) to obtain the actual TCR value. Figure 5e shows the linear behavior of the *R*–*T* relationship fitting the parameters of the slope of 0.0865 and the intercept of 19.9. The TCR, *α*, of the RTD sensor was computed by taking the slope/intercept, which is 0.00435. This value is within the range of the NiFe’s TCR. Either fitted line or calibrated *R*–*T* data is used in monitoring the temperature of the SS PC simply from reading the resistance value of the RTD sensor.

### 3.4. In-Line Injection Performance of the Printed PCs

The proposed printed PC works by sorption trapping followed by the thermal desorption of high surface area adsorbents. Before testing the device for in-line injection performance, we need to ensure that the adsorbents can be heated to the target desorption temperature, which is about 180 °C for Hayesep D. We also note that the temperature of the membrane heater can be monitored via the RTD sensor, but not the PC itself. Once the heater is attached to the PC, the overall thermal mass increases significantly, slowing down the heating rate and the highest achievable temperature. In addition, a carrier gas flowing inside the PC further reduces the device’s heating rate and maximum temperature. Therefore, a series of experiments was conducted to determine the appropriate membrane temperature that provides sufficient heating power for the PC to reach 180 °C for a given flow rate. Our experiments showed that when the membrane heater was heated to approximately 350 °C, the center region of the PC (measured by a thermocouple probe) only reached 183 °C for a constant helium flow rate of 0.5 mL/min.

**Figure 6:**
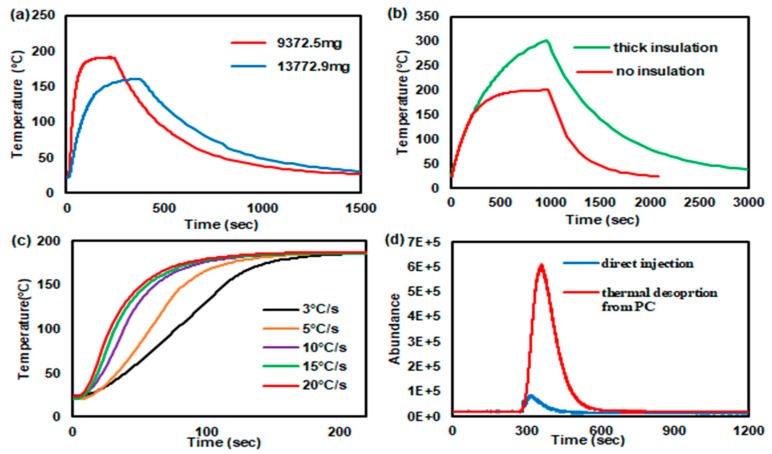
Temperature profiles of the SS PC (SS316/BN) (**a**) with different thermal mass (no insulation); (**b**) with and without thermal insulation (9372.5 mg sample); (**c**) a family of the temperature profiles of the SS PC with different heat ramping rates; (**d**) the mass spectrometer responses for the direct splitless injection of 0.5 mL of 0.6% headspace toluene (blue line) and for the thermal desorption peak of toluene after sampling of 10 mL of 0.6% headspace toluene into the SS PC.

A heating rate, i.e., how rapidly the PC reaches the target temperature, depends on the thermal mass, applied power, and level of thermal insulation. A smaller thermal mass is more beneficial in terms of thermal performance metrics such as ramp-up time and powder consumption. To reduce the thermal mass, the outer diameter of the SS316/BN PC was turned from 9.3 mm to 7.69 mm, reducing the mass from 13773 mg to 9373 mg. We also tried to print SS PCs with thinner walls, but the unsintered parts became too fragile to be handled during the powder removal and curing steps. Figure 6a shows the temperature profiles as a function of time when the membrane heater was heated to the same target temperature (and same ramping rate) for 3 min to each PC of two different thermal masses. The temperature of the SS PC with smaller thermal mass was raised to 180 °C in about 85 seconds, while the larger PC reached the maximum temperature of 160.5 °C in about 370 seconds. Therefore, for the rest of the study, we present the experimental results of the PC with smaller thermal mass.

Another important factor in heater/PC performance is thermal isolation. There are three main heat losses in the PC: convective and radiative losses around the surface area of the membrane heater, and conductive loss to the fluidic connectors at both ends. PEEK fittings were used to minimize the conductive loss, and further improvement in thermal isolation to the sides is difficult achieve. Convective and radiative losses around the membrane heater can be significantly reduced by wrapping a high-temperature insulation mat around the PC. A thin aluminum foil was sandwiched between two layers of the insulation mat to further prevent any radiative loss. Figure 6b shows the different temperature responses of the same PC with different thermal insulation conditions; 4.5 W was applied to the heater for both conditions. It can be observed that the center region of the SS PC heats up to around 180 °C in 15 min without insulation, while the temperature of the center region rises to 320 °C in the same time. This means that thermal insulation allows the PC to reach the target temperature more rapidly or with less power consumption. However, the insulation layer impedes a cooling process, which is represented by the slower decay of the temperature in Figure 6b. This means that one has to wait longer before the next run can be initiated. Therefore, a decision remains to be made of whether thermal insulation should be integrated depending on the operational requirements (power consumption versus speedy recovery).

The center cavity of the device was filled with solid adsorbents (HayeSep D, Sigma Aldrich) to trap volatile compounds (toluene) for PCs (see Supplementary Information Figure S1). When adsorbent materials such as Hayesep D are subjected to the temperature beyond the suggested level (~290 °C), they start to degrade and lose adsorption capacity. Therefore, an open-loop control of temperature (i.e., the PC temperature simply controlled by the level of constantly applied power) may be inappropriate for the long-term use of the porous polymer adsorbents. An integrated RTD sensor can maintain the PC temperature at the target level by means of feedback control. Figure 6c shows the temperature profiles of the SS PC with various heating rates from 3 °C/s to 20 °C/s. The highest heating rate (20 °C/s) can have the absorbents reach the desorption temperature (180 °C) in about 100 s. The other heating rates of 15 °C/s, 10 °C/s, 5 °C/s, and 3 °C/s took 110 s, 117 s, 136 s, and 166 s to reach 180 °C, respectively. For the desorption testing, we chose 20 °C/s for heating rate for a fast response. In the future, we can further reduce the thermal mass of sample through changing the design or machining out a greater mass of the sample, which is also an advantage of 3D printing technology for fabricating PCs.

Finally, the feasibility of using the PC as an inline injector is demonstrated using toluene as a model volatile organic compound. When 0.5 mL of 0.6% diluted toluene from the headspace in a sampling bag was directly introduced to the GC/MS via splitless injection, the relatively small peak was observed (blue line) in Figure 6d. To enhance the detector’s response, 10 mL of the same diluted toluene (0.6%) was sampled by the PC. We observed the toluene peak during the sampling process, meaning that the adsorbents were fully charged. Upon heating of the PC to the target desorption temperature (for Hayesep D, 180 °C), the trapped toluene was released in a relatively short time, and the concentrated pulse was delivered to the detector by the carrier gas flowing through the PC. The red line in Figure 6d shows the concentrated toluene peak after thermal desorption (see Supplementary Information Figure S6 for the toluene verification by the mass spectrometer). The area under the curve in the chromatogram is related to the amount of the detected molecules. The ratio of the peak areas for both signals was estimated to be 14.2, which is less than the sampling volume ratio of 10/0.5 = 20. It can be attributed that some toluene may have been lost during the purging step and by condensation inside the connectors/tubing. In the end, if the PC is placed in front of a gas detector, it can be used to trap VOCs of low concentration and make an injection of more concentrated analytes at any desirable time without resorting to valve operation.

## 4. Conclusions

Recent advances in additive manufacturing (AM) or metal 3D printing technology have enabled the fabrication of freeform metallic parts with intricate features. However, metallic parts with complex internal features for microchannel/microreactor applications are still difficult to be achieved with the more common AM technologies such as selective laser sintering and selective laser melting due to difficulties associated with powder removal from the internal cavities. Binder jet printing (BJP), one of the oldest AM methods, has several advantages, including easier powder removal, less residual stress in the final parts, and the capability of producing full-density metal components with internal microchannels with the addition of BN. The key challenge for BJP is controlling the porosity, as large interconnected pores may case leaking through the walls of the parts and are not suitable for fluidic applications. In this paper, it was shown that the addition of sintering additives such as boron nitride (BN) can facilitate the sintering of stainless steel (SS) to create near full-density parts. A reduction in porosity by BN was visualized and quantified via image analysis. A proof-of-the-concept preconcentrator (PC) device was built and tested as volatile organic compounds (VOC) sampling and inline injection to be used in conjunction with gas chromatography and mass spectrometry. Unlike miniature or microscale PCs commonly produced with silicon, the printed SS PCs can be easily machined to be compatible with commercially available compression fittings, which enables long-term, robust fluidic connections that are suitable for medium to high-temperature applications. It has been shown that the micro-PC filled with the Hayesep D adsorbent can trap low-concentrated toluene and enhance the detector signal by more than 10 times. The next step is to further reduce the thermal mass of the PC by optimizing the BJP process, allowing for faster heating rates, sharpening the desorption peak, and improving the overall detection response.

## Figures and Tables

**Table 1:** A comparison of the chemical composition of SS316 between the bulk value and 3D printed part (electron dispersive x-ray spectroscopy (EDS) data analysis can be referred to Supplementary Information Figure S2).

	Fe	Cr	Ni	C	Mo	Mn	Si
Bulk SS316	bal.	16~18	10~14	0.08 max	2~3	2 max	0.75 max
EDS measured (SS316 + BN)	61.3	16	11.3	6.9	2.1	1.6	0.7

(Note) The numbers are in percent by weight.

**Table 2:** A comparison of the chemical composition of SS316/0.25%BN sample obtained from the X-ray photoelectron spectroscopy (XPS) data analysis (see Supplementary Information Figure S3).

	B 1s	C 1s	O 1s	Ca 2p3	Fe 2p	Cr 2p	Ni 2p	Mo 3d5	Mn
Run 1	1.6	14.1	30.4	3.1	22.0	22.0	0	0	6.8
Run 2	0	3.4	5.1	0	56.2	13.0	13.5	8.9	0

(Note) The numbers are in percent by weight.

## Supplementary Materials

The following are available online at https://www.mdpi.com/1424-8220/19/12/2748/s1.
