# Supplementary material for: A Binder Jet Printed, Stainless Steel Preconcentrator as an In-Line Injector of Volatile Organic Compounds
Source: Sensors (Basel). 2019 Jun 19;19(12):2748. doi: 10.3390/s19122748 (PMC6630219; doi:10.3390/s19122748)
Supplement: Supplementary file 1 [file sensors-19-02748-s001.pdf]

Supplementary Information

A binder jet printed, stainless steel preconcentrator as an in-line injector of volatile organic compounds

Xiaolu Huang[1], Tyler Bauder[1], Truong Do[1], Hawke Suen[1], Connor Boss[2], Patrick Kwon[1], and Junghoon Yeom[1]*

1. Department of Mechanical Engineering, Michigan State University, East Lansing, MI 48824
2. Department of Electrical Engineering, Michigan State University, East Lansing, MI 48824

*** Email: jyeom@egr.msu.edu**

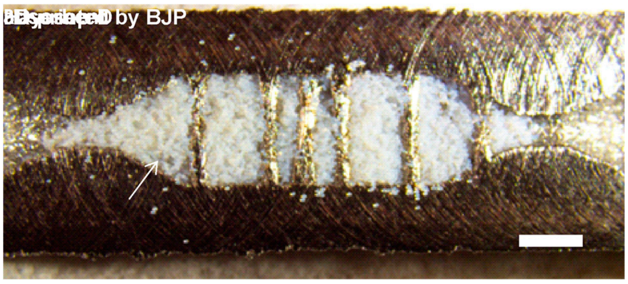

Figure S1. A photograph of the cross-sectional view of the SS PC (SS316 and BN) filled with the porous polymer adsorbent, Hayesep D. Scale bar = 3 mm.

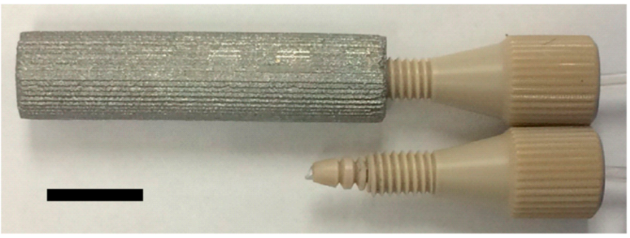

Figure S2. A photograph of the SS PC (with BN) connected with the commercially available PEEK fitting (for 1/16” tubing, IDEX Health & Science). Scale bar = 1 cm.

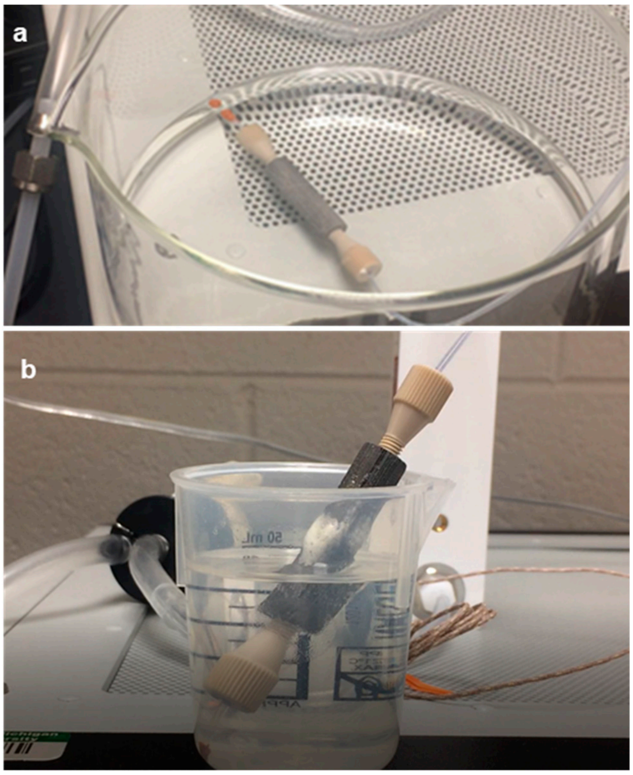

Figure S3. Leak test of air applied to the dead end through the SS PCs with a) SS316 with BN and b) SS316 only. The PCs were immersed in an IPA solution.

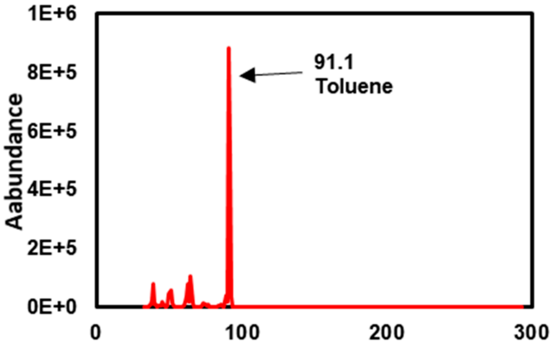

Figure S4. Mass-spectrometer signal of the desorbed analyte from the SS PC, confirming that the peak is associated with toluene.

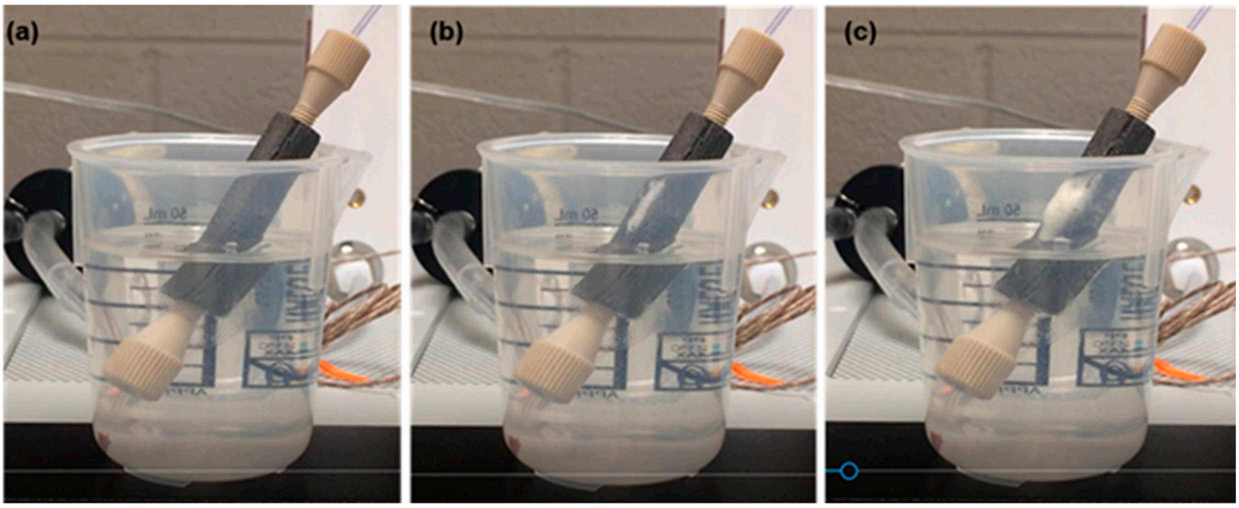

Video clip of leak test for SS316 only sample: when N_2 go through the SS316 only sample with small pressure (~64kPa), a big leak (bubbles) was observed (a-c) on the surface of the sample wall, meaning high porosity of the sample.

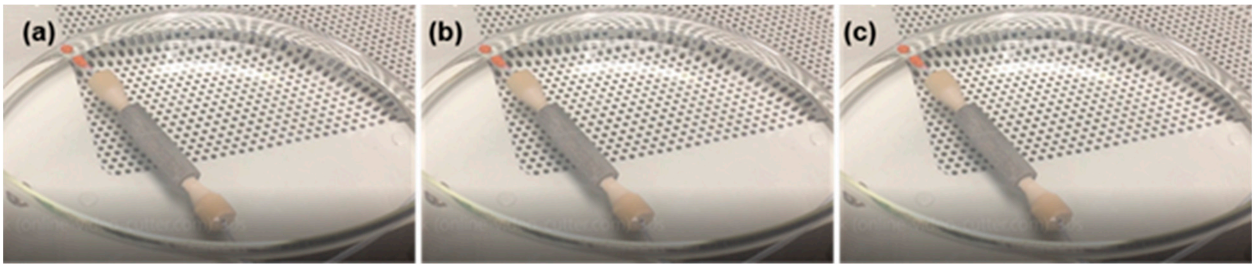

Video clip of leak test for SS316/BN sample: 500kPa pressure of N_2 was flowing to dead end PC (SS316 with BN) system and no leak (bubble) was found in the 3DP sample and connection between sample with tubing. This leak-tight results attribute to the near full dense structure when BN was used to improve the dense of sample after sintering.
